# Effectiveness of irinotecan plus trabectedin on a desmoplastic small round cell tumor patient-derived xenograft

**DOI:** 10.1242/dmm.049649

**Published:** 2023-06-14

**Authors:** Valentina Zuco, Sandro Pasquali, Monica Tortoreto, Stefano Percio, Valentina Doldi, Marta Barisella, Paola Collini, Gian Paolo Dagrada, Silvia Brich, Patrizia Gasparini, Marco Fiore, Michela Casanova, Anna Maria Frezza, Alessandro Gronchi, Silvia Stacchiotti, Andrea Ferrari, Nadia Zaffaroni

**Affiliations:** ^1^ Molecular Pharmacology Unit, Department of Experimental Oncology, Fondazione IRCSS Istituto Nazionale dei Tumori, 20133 Milan, Italy; ^2^ Sarcoma Service, Department of Surgery, Fondazione IRCSS Istituto Nazionale dei Tumori, 20133 Milan, Italy; ^3^ Soft Tissue Tumor Pathology Unit, Department of Advanced Diagnostics, Fondazione IRCSS Istituto Nazionale dei Tumori, 20133 Milan, Italy; ^4^ Epigenomics and biomarkers of solid tumors, Department of Experimental Oncology, Fondazione IRCSS Istituto Nazionale dei Tumori, 20133 Milan, Italy; ^5^ Pediatric Oncology Unit, Department of Cancer Medicine, Fondazione IRCSS Istituto Nazionale dei Tumori, 20133 Milan, Italy; ^6^ Medical Oncology Unit 2, Department of Cancer Medicine, Fondazione IRCSS Istituto Nazionale dei Tumori, 20133 Milan, Italy

**Keywords:** Desmoplastic small round cell tumor, Patient-derived xenograft, Chemotherapy

## Abstract

This study exploited a novel patient-derived xenograft (PDX) of desmoplastic small round cell tumor (DSRCT), which reproduces histomorphological and molecular characteristics of the clinical tumor, to assess the activity of cytotoxic and targeted anticancer agents. Antitumor effect was moderate for doxorubicin, pazopanib and larotrectenib [maximum tumor volume inhibition (max TVI), 55-66%], while trabectedin had higher activity (max TVI, 82%). Vinorelbine, irinotecan and eribulin achieved nearly complete tumor growth inhibition (max TVI, 96-98%), although tumors regrew after the end of treatment. The combination of irinotecan with either eribulin or trabectedin resulted in complete responses, which were maintained until the end of the experiment for irinotecan plus trabectedin. Irinotecan-based combinations nearly abrogated the expression of proteins of the G2/M checkpoint, preventing cell entrance in mitosis, and induced apoptotic and necroptotic cell death. Consistently, irinotecan plus trabectedin resulted in reprogramming of DSCRT transcriptome, with downregulation of E2F targets, G2/M checkpoint and mitotic spindle gene sets. This study emphasizes the importance of patient-derived preclinical models to explore new treatments for DSRCT and fosters clinical investigation into the activity of irinotecan plus trabectedin.

## Teaser

**Summary:** This study shows the effectiveness of irinotecan, eribulin, trabectedin and their combination in a patient-derived xenograft model of desmoplastic small round cell tumor, a rare and incurable malignancy.

## INTRODUCTION

Desmoplastic small round cell tumor (DSRCT) is an ultra-rare sarcoma characterized by the specific chromosomal translocation t(11:22)(p13;q12) that leads to the fusion of the transcriptional regulatory domain of EWSR1 to the DNA-binding domain of WT1, resulting in the oncogenic *EWSR1::WT1* gene fusion (Gerald et al., 1991; Ladanyi and Gerald, 1994). The vast majority of patients affected by DSRCT are children and young adults with a male preponderance (Gerald et al., 1998). Advanced disseminated disease is commonly detected at diagnosis, with large abdominal masses consisting of multiple peritoneal nodules and extensive peritoneal seeding. Extraperitoneal metastases to lymph nodes, liver and lungs may also be present at disease onset (Gerald et al., 1998). Currently available treatment options include a combination of surgery, chemotherapy and radiotherapy (Mir et al., 2018). These approaches have limited efficacy, and most patients eventually experience recurrence and die from the disease 17-25 months after diagnosis (Bulbul et al., 2017). New anticancer agents have been tested, including irinotecan (Rosoff and Bayliff, 1999), which can be combined with other cytotoxic agents, such as ifosfamide, vincristine and actinomycin-D, exploiting its favorable toxicity profile to enhance drug dose and schedule density (Bisogno et al., 2021; Ferrari et al., 2022a). Other treatments showed some effectiveness in patients who experienced disease recurrence, including the cytotoxic agents trabectedin (Frezza et al., 2014b; Verret et al., 2017; Brunetti et al., 2014; Lopez-Gonzalez et al., 2011) and eribulin (Emambux et al., 2017) or multikinase inhibitors (Frezza et al., 2014a; Italiano et al., 2013).

The low incidence of DSRCT and the wide spectrum of clinical presentations challenge the ability to conduct prospective clinical studies, which ultimately hamper therapeutic progress for this tumor. This is mirrored by the paucity of DSRCT experimental models able to properly recapitulate tumor biology and response to treatment. Indeed, in addition to the widely available JN-DSRCT-1 cell line, first described by Nishio et al. in 2002 (Nishio et al., 2002), four additional tumorigenic DSRCT cell lines and three patient-derived xenografts (PDXs) have only recently been developed (Smith et al., 2022; Slotkin et al., 2021; Ogura et al., 2021).

An advantage of PDXs compared with cell line-derived xenografts is their ability to better predict tumor response to therapeutic agents and provide insights into drug scheduling (Hamacher and Bauer, 2017; Bleijs et al., 2019). We previously demonstrated consistency between preclinical data obtained in PDXs of other ultra-rare soft-tissue sarcoma histologies, such as solitary fibrous tumor (Stacchiotti et al., 2017) and epithelioid sarcoma (Stacchiotti et al., 2019b), and clinical results concerning the activity of several cytotoxic, antiangiogenic and epigenetic agents. We translated preclinical data into the design of new successfully conducted prospective clinical trials (Martin-Broto et al., 2019, 2020; Stacchiotti et al., 2019a).

This study describes a novel DSRCT PDX model (DSRCT-1) that we exploited to comparatively assess the activity of cytotoxic and targeted agents of potential interest for this malignancy and clinically available for the treatment of sarcomas. In order to strengthen evidence from early clinical reports (Bisogno et al., 2021; Ferrari et al., 2022a; Frezza et al., 2014b; Verret et al., 2017; Brunetti et al., 2014; Lopez-Gonzalez et al., 2011; Emambux et al., 2017), we examined the anticancer effectiveness of agents such as irinotecan, eribulin and trabectedin in this PDX model, observing the highest activity for the irinotecan-trabectedin combination.

## RESULTS

### The DSRCT-1 PDX model recapitulates histomorphological and molecular characteristics of the paired clinical tumor

**Fig. 1.**
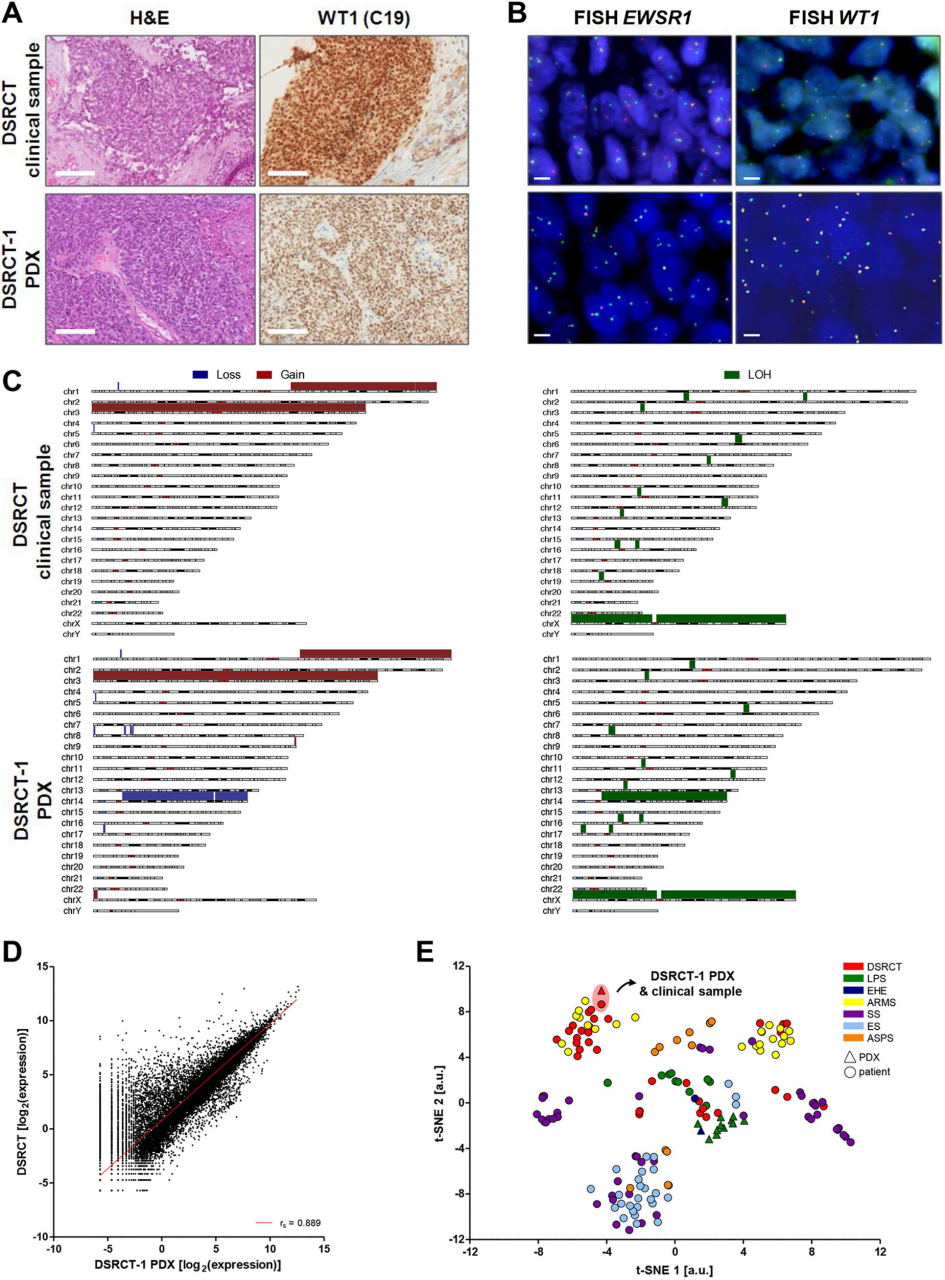
**Characterization of a patient-derived xenograft (PDX) model of desmoplastic small round cell tumor (DSRCT).** (A,B) Representative pictures of the DSRCT clinical sample and corresponding PDX model (DSRCT-1). (A) Histology was assessed on Hematoxylin and Eosin (H&E)-stained slides. WT1 (C19) expression was detected at the protein level by WT1 immunostaining. Scale bars: 100 µm. (B) Evaluation of *EWSR1* (*EWS*) and *WT1* status by fluorescence *in situ* hybridization (FISH); split green and orange signals indicate rearranged alleles. Scale bars: 10 µm. (C) Genomic profile of DSRCT clinical tumor (top row) and PDX (bottom row). Loss (blue) and gain (red) are depicted in the left column. Loss of heterozygosity (LOH; green) is shown in the right column. (D) Scatterplot of significant correlation between the transcriptome of the clinical tumor and the paired PDX (Spearman correlation coefficient *r_s_*=0.889). (E) Scatterplot of t-distributed stochastic neighbor embedding (t-SNE) analysis of metadataset samples. ARMS, alveolar rhabdomyosarcoma; ASPS, alveolar soft part sarcoma; a.u., arbitrary units; EHE, epithelioid hemangioendothelioma; ES, epithelioid sarcoma; LPS, liposarcoma; SS, synovial sarcoma.

The histomorphology of the DSRCT-1 PDX was consistent with that of the paired clinical tumor. Both tumors were characterized by the presence of necrosis, surrounded by viable tumor cells and cells exhibiting features of apoptosis (Fig. 1A). Immunohistochemistry (IHC) confirmed positivity for WT1 expression, and fluorescence *in situ* hybridization (FISH) analysis revealed the presence of the pathognomonic t(11:22)(p13;q12) *EWSR1::WT1* translocation (Fig. 1B).

Genomic analysis of the clinical tumor and PDX revealed gains in chromosome 1q and chromosome 3, as well as a loss of heterozygosity (LOH) in chromosome 3 involving the 3p21.1 band, which includes the *BAP1* gene, in both tumors (Fig. 1C). Loss of chromosome 14 was identified in the PDX and not in the clinical tumor, suggesting a possible clonal selection in the PDX (Fig. 1C).

Interestingly, whole-transcriptome sequencing revealed a highly significant correlation in gene expression profile between the clinical tumor and the PDX (*r_s_*=0.889; *P*<0.001) (Fig. 1D). The transcriptomic profiles of the PDX and clinical samples clustered together with those of DSRCT clinical samples analyzed in other studies (Fig. 1E) (GSE220172 and GSE90904; Smith et al., 2022). Distribution of DSRCT likely depicts heterogeneity within and between the two analyzed studies (GSE220172 and GSE90904; Smith et al., 2022) as well as in the PDXs from our library. These may include differences in disease extent, chemotherapy schedules, and the profiling of untreated biopsy or surgical samples after chemotherapy. The clustering of DSRCT with alveolar rhabdomyosarcoma (ARMS) may reflect the round cell morphology and the simple karyotype, although with a different translocation, of these tumors. Moreover, the comparison of our paired DSRCTs with clinical samples and PDX models of other sarcoma histologies highlighted the distinctive transcriptomic profile of DSRCT (Fig. 1E). These findings suggest that our PDX model is potentially highly valuable to represent the clinical DSRCT in preclinical pharmacology studies.

### The irinotecan-trabectedin combination shows the highest antitumor activity

**Fig. 2.**
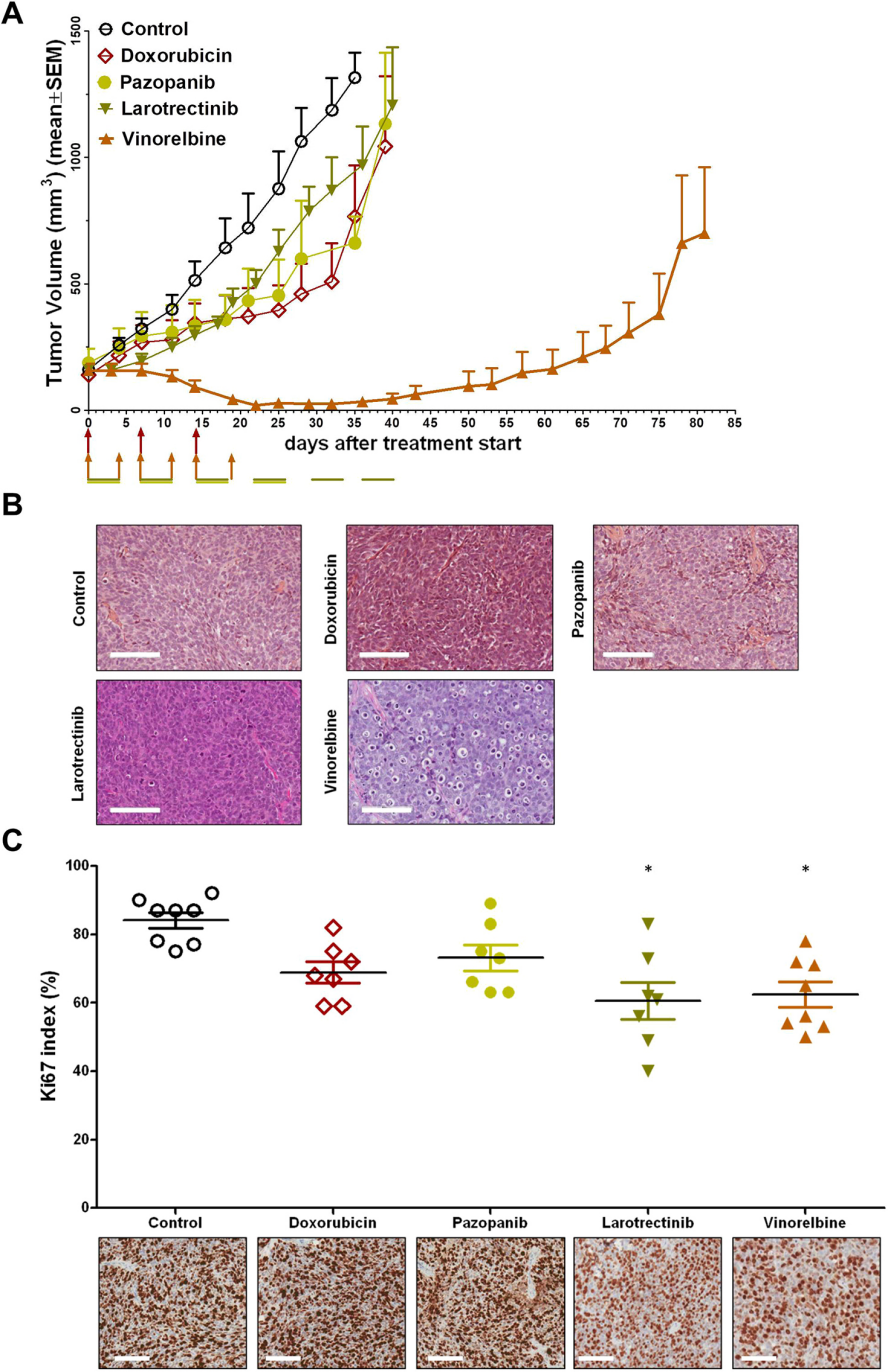
**Antitumor activity of doxorubicin, pazopanib, larotrectinib or vinorelbine as single agents.** (A) Growth curves report the average tumor volume (±s.e.m.) in control and drug-treated animal groups (six mice/group). The arrows indicate when drugs were administered. (B) Histomorphological evaluation of tumors obtained from untreated (Control) and drug-treated mice. Scale bars: 100 µm. (C) Ki67 immunostaining of tumors obtained from untreated (Control) and drug-treated mice (bottom) and quantification of Ki67 index (top). Symbols in the top panel represent counted fields. Histomorphological analysis and Ki67 immunostaining were performed on tumors excised from mice after the first round of treatment with each drug. Scale bars: 100 µm. *P<0.05, Kruskal–Wallis test.

To assess the ability of our DSRCT-1 PDX model to reproduce the activity of anticancer drugs available for the clinical management of DSRCT patients, we first examined the efficacy of single-agent doxorubicin, pazopanib and vinorelbine. Because the *EWSR1::WT1* gene fusion activates the transcription of *NTRK3* (Ogura et al., 2021), we also tested the NTRK inhibitor larotrectenib. A moderate antitumor effect was observed following treatment with doxorubicin, pazopanib or larotrectenib, with maximum tumor volume inhibition (max TVI) ranging from 55% to 66%; vinorelbine induced almost complete inhibition of tumor growth (max TVI, 98%), although tumors started to regrow ∼20 days after the end of treatment (Fig. 2A, Table 1). Histopathological evaluation of tumors excised from mice after the first round of individual treatment with doxorubicin, pazopanib or larotrectinib revealed no appreciable differences compared to tumors from untreated mice, whereas tumors treated with vinorelbine showed features of necrotic cell death (Fig. 2B). The proliferation rate, as detected by the Ki67 (also known as MKI67) index (Nielsen et al., 2021), in the same tumors indicated a limited reduction (∼15-20%) in the percentage of proliferating cells after exposure to the different drugs (Fig. 2C), which reached statistical significance (*P*<0.05) for larotrectinib and vinorelbine.

**Table 1.**
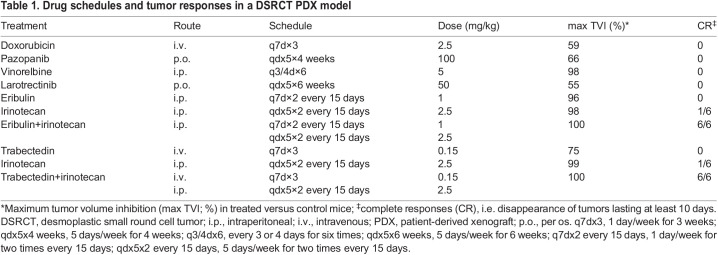
Drug schedules and tumor responses in a DSRCT PDX model

**Fig. 3.**
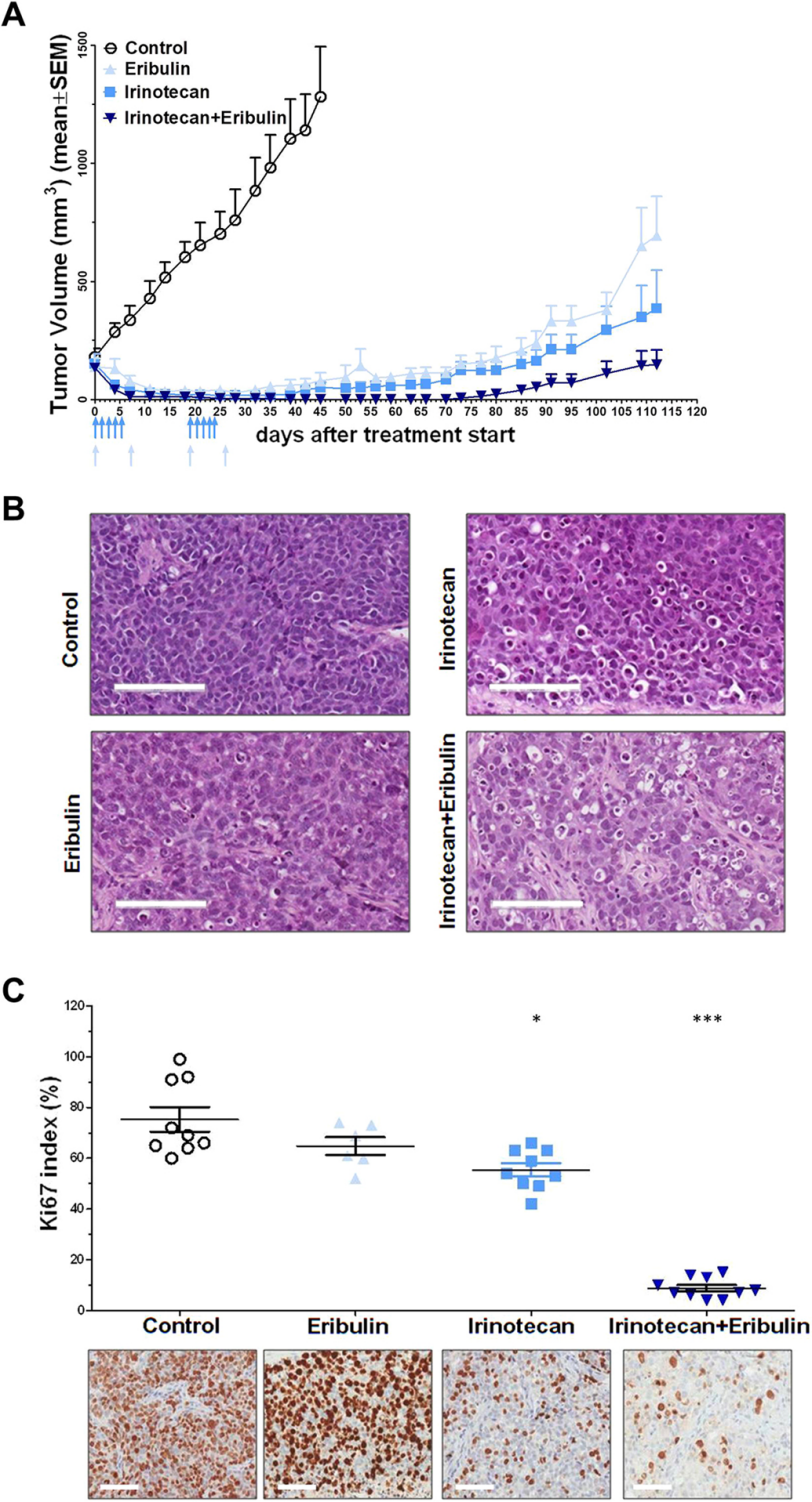
**Antitumor activity of irinotecan or eribulin as single agents or in combination.** (A) Growth curves report the average tumor volume (±s.e.m.) in control and drug-treated animal groups (six mice/group). (B) Histomorphological evaluation of tumors obtained from untreated (Control) and drug-treated mice. Scale bars: 100 µm. (C) Ki67 immunostaining of tumors obtained from untreated (Control) and drug-treated mice (bottom) and quantification of Ki67 index (top). Symbols in the top panel represent counted fields. Histomorphological analysis and Ki67 immunostaining were performed on tumors excised from mice after the first round of individual or combined treatment. Scale bars: 100 µm. *P<0.05, ***P<0.001, Kruskal–Wallis test.

To identify novel active combinations, we also assessed the activity of irinotecan and eribulin, singly administered and combined, in our PDX model. Both drugs induced almost complete tumor growth inhibition, with a max TVI of 98% for irinotecan (with one mouse experiencing a complete response lasting 34 days) and 96% for eribulin (Fig. 3A, Table 1). However, tumor regrowth started ∼20 days after the end of treatment for each drug. Interestingly, the combined irinotecan-eribulin treatment reached 100% max TVI, with all mice showing a complete response that was appreciable until 46 days after the last treatment (Fig. 3A, Table 1). Histopathological evaluation of tumors excised from mice after the first round of individual or combined treatment showed a higher presence of apoptotic cells, necrosis and perivascular sclerojalinosis after irinotecan plus eribulin compared to that after individual drugs (Fig. 3B; Fig. S1A). Consistently, Ki67 analysis showed a statistically significant (*P*<0.001) reduced percentage of proliferating cells in irinotecan plus eribulin-treated tumors compared to that in untreated tumors (Fig. 3C).

**Fig. 4.**
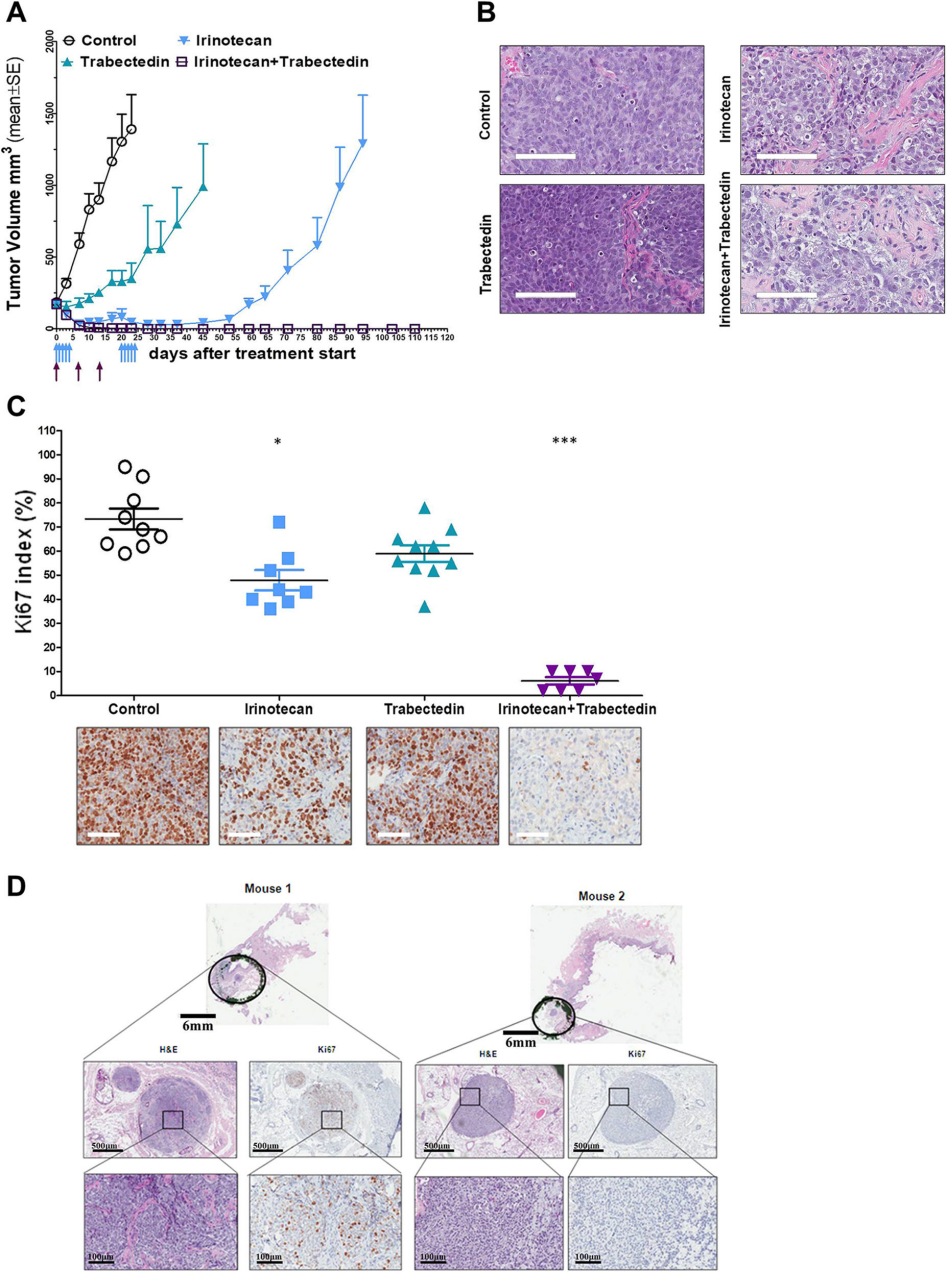
**Antitumor activity of irinotecan or trabectedin, as single agents or in combination.** (A) Growth curves report the average tumor volume (±s.e.m.) in control and drug-treated animal groups (six mice/group). (B) Histomorphological evaluation of tumors obtained from untreated (Control) and drug-treated mice. Scale bars: 100 µm. (C) Ki67 immunostaining of tumors obtained from untreated (Control) and drug-treated mice (bottom) and quantification of Ki67 index (top). Symbols in the top panel represent counted fields. Histomorphological analysis and Ki67 immunostaining were performed on tumors excised from mice after the first round of individual or combined treatment. Scale bars: 100 µm. (D) H&E staining and Ki67 immunostaining in residual tumor cells detected at the end of the experiment (i.e. 86 days after the last treatment) in tumor specimens from two mice. Ki67 index was ∼40% in one specimen (Mouse 1); no Ki67-positive cells were detected in the other specimen (Mouse 2). Scale bars: 6 mm, 500 µm and 100 µm. *P<0.05, ***P<0.001, Kruskal–Wallis test.

In a further experiment, we assessed the activity of the trabectedin-irinotecan combination. As individual agents, trabectedin achieved 82% max TVI, while irinotecan induced almost complete abrogation of tumor growth (99% max TVI), although a steeper increase in tumor volume was detected after the end of treatment than that after the previous experiment. The addition of irinotecan to trabectedin enhanced max TVI to 100%, with complete response in all mice, which was still appreciable at the end of the experiment (i.e. 86 days from the last treatment), with no evidence of tumor regrowth (Fig. 4A, Table 1). Histopathological evaluation of tumors explanted from mice after the first round of individual or combined treatment showed increased perivascular sclerojalinosis, necrosis and apoptosis after irinotecan alone or irinotecan plus trabectedin (Fig. 4B; Fig. S1). Remarkably, this combination resulted in the most relevant post-treatment changes. Tumors from mice exposed to the irinotecan-trabectedin combination were also characterized by the lowest Ki67 index, with a 90% reduction compared to that in tumors from untreated mice (*P*<0.001; Fig. 4C).

To assess whether the absence of palpable tumor in mice treated with the irinotecan-trabectedin combination was associated with a pathological complete remission, we performed histological analyses of animals sacrificed at the end of the experiment. Residual tumor cells were detected in only two of four evaluable tissue specimens, along with treatment-induced necrosis and sclerojalinosis. Proliferation rate, as measured by the Ki67 index, was ∼40% in one tumor (Mouse 1); no evidence of proliferating cells was observed in the other tumor (Mouse 2), in which histiocytosis was also evident (Fig. 4D).

Concerning drug-induced toxicities, all individual and combined treatments did not cause weight loss (Fig. S2) or toxic deaths in mice.

### The irinotecan-trabectedin combination induces cell cycle perturbations and programmed cell death

**Fig. 5.**
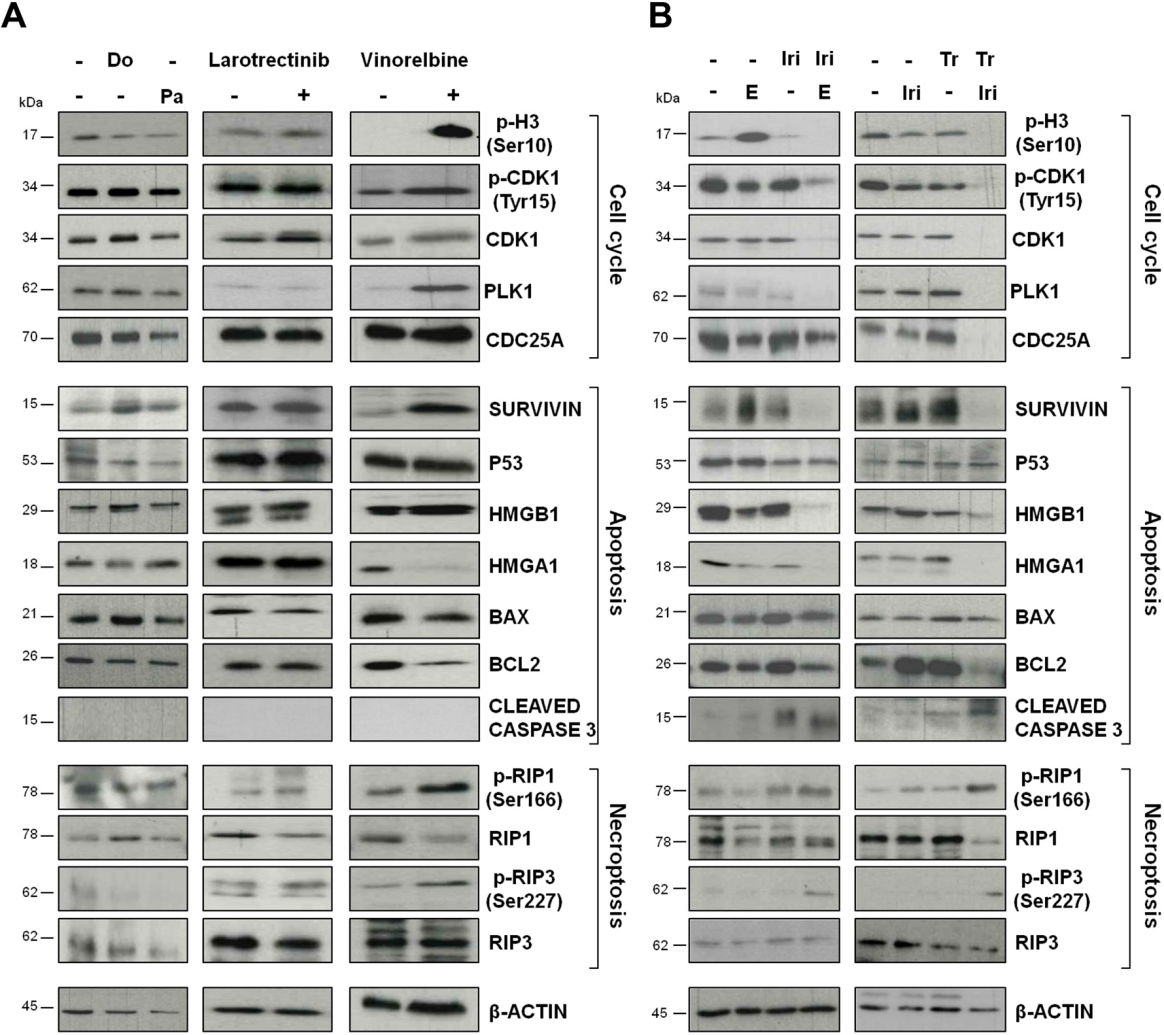
**Effects of irinotecan or trabectedin, as single agents or in combination, on the expression of proteins related to cell cycle, apoptosis and necroptosis.** (A) Doxorubicin (Do), pazopanib (Pa), larotrectinib and vinorelbine were evaluated as single agents. (B) Irinotecan (Iri), eribulin (E) and trabectedin (Tr) were evaluated as single agents or in combination. Western blot analysis was performed on tumors excised from mice after the first round of individual or combined treatment. Cropped images of selected proteins are shown in the different blots.

Western blot analysis of tumors explanted from mice after the first round of individual or combined drug treatment indicated that both irinotecan-based combinations almost completely abrogate the expression of proteins involved in the G2/M checkpoint, such as CDK1 and its active form phospho (p)-CDK1 (Tyr15), CDC25A and PLK1 (Zuco et al., 2015; Smith et al., 2020), thus preventing cell entrance in mitosis, as also suggested by the reduced expression of the specific mitotic marker p-H3 (Ser10) (Perez-Cadahia et al., 2009) (Fig. 5; Fig. S3A,B). Of note, an accumulation of DSRCT cells in G2 phase following trabectedin exposure was previously reported (Uboldi et al., 2017). Western blot results were also consistent with IHC data indicating a significantly reduced Ki67 index in tumors exposed to each irinotecan-based combination (Fig. 3C, Fig. 4C). No appreciable inhibition of the expression/activation of G2/M-related proteins was induced by individual treatment with irinotecan, eribulin, trabectedin, doxorubicin, pazopanib or larotrectinib. Conversely, consistent with the induction of a mitotic block, vinorelbine caused a marked accumulation of p-H3 (Ser10) and PLK1 (Fig. 5). This finding is in keeping with the modest decrease in Ki67 index observed after treatment with vinorelbine, based on the notion that Ki67 protein is maximally expressed in G2 phase and mitosis (Uxa et al., 2021).

An apoptotic response following exposure to both irinotecan-based combinations was also suggested by a treatment-induced decrease in the expression of the antiapoptotic proteins survivin and BCL2, as well as of other proteins known to counteract programmed cell death, such as HMGA1 (Ma et al., 2020) and HMGB1 (Sharma et al., 2022; He et al., 2012), which was paralleled by the presence of cleaved caspase-3 (Fig. 5). Remarkably, *BIRC5*, the gene coding for survivin, was downregulated by the combination of irinotecan plus trabectedin (Fig. S4). Moreover, the accumulation of p-RIP1 (also known as p-RIPK1) (Ser166) and p-RIP3 (also known as p-RIPK3) (Ser227) in tumors exposed to irinotecan-based combinations, as well as to single-agent vinorelbine, suggests treatment induction of necroptosis, a type of programmed cell death with necrotic morphology (Gong et al., 2019), which is consistent with the results of histomorphological analysis of tumors explanted from treated mice (Fig. 2B, Fig. 3B).

**Fig. 6.**
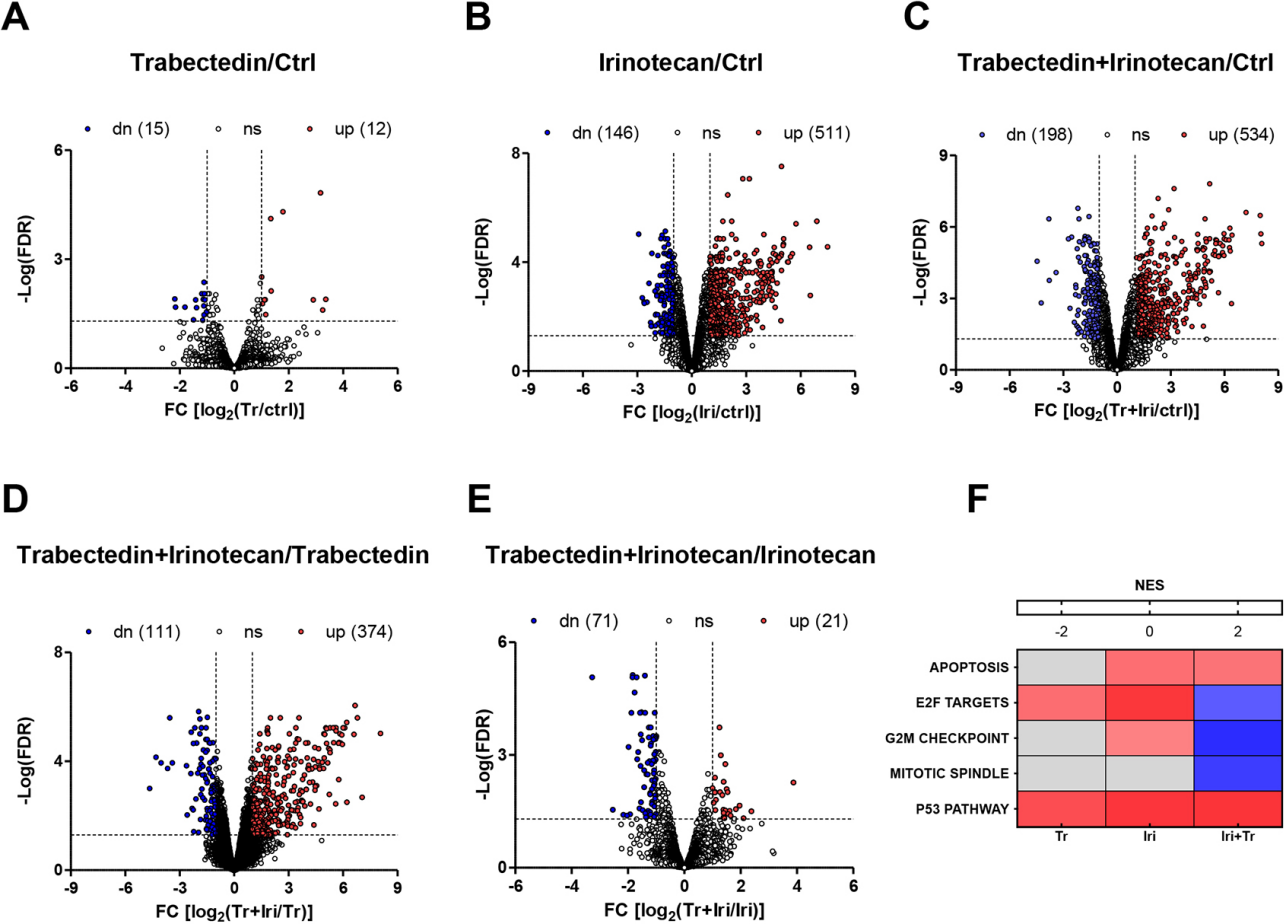
**Effects of irinotecan or trabectedin, as single agents or in combination, on PDX transcriptome profile.** (A-E) Volcano plots showing the transcriptional changes induced by different treatments: individual and combined treatment versus untreated controls (ctrl) (A-C); trabectedin-irinotecan combination versus individual treatment (D,E). An absolute log2(FC)>1 and an FDR<0.05 were used to select significantly different expressed genes (upregulated in red and downregulated in blue). (F) Heatmap of the normalized enrichment score (NES) for deregulated hallmark gene sets in DSRCT PDXs treated with either trabectedin, irinotecan or their combination, with respect to untreated PDX. Gray boxes represent non-statistically significant gene sets (FDR<0.05). RNA-sequencing analyses were performed on tumors obtained from three mice for each experimental group. dn, downregulated; FC, fold change, FDR, false discovery rate; Iri, irinotecan; ns, not significant; Tr, trabectedin; up, upregulated.

To gain insights into the molecular mechanisms underpinning the effects induced by trabectedin and irinotecan, as single agent and in combination, RNA sequencing was performed in tumors excised from mice after the first round of individual or combined treatment (Fig. 6A-E). Single-agent irinotecan induced more pronounced alteration of the transcriptomic profile than did trabectedin (Fig. 6A,B), although the most important transcriptomic reprogramming was induced by the combination of irinotecan plus trabectedin (Fig. 6C-E). Consistently, tumors treated with the same drug schedule clustered together, as visualized using the t-distributed stochastic neighbor embedding (t-SNE) method (Fig. S5). The analysis of hallmark gene set enrichment showed an upregulation of gene sets related to apoptosis and the P53 pathway after individual and combined drug treatments (Fig. 6F). The irinotecan-trabectedin combination, but not each drug as a single agent, resulted in a marked downregulation of E2F targets, G2/M checkpoint and mitotic spindle gene sets (Fig. 6F). The *AURKA*, *CDC25B* and *PLK1* genes were included in these gene sets and were consistently downregulated after treatment with irinotecan plus trabectedin (Fig. S4). Indeed, this is in agreement with western blot results suggesting the arrest of tumor cells in the G2 phase of the cell cycle (Fig. 5B).

**Fig. 7.**
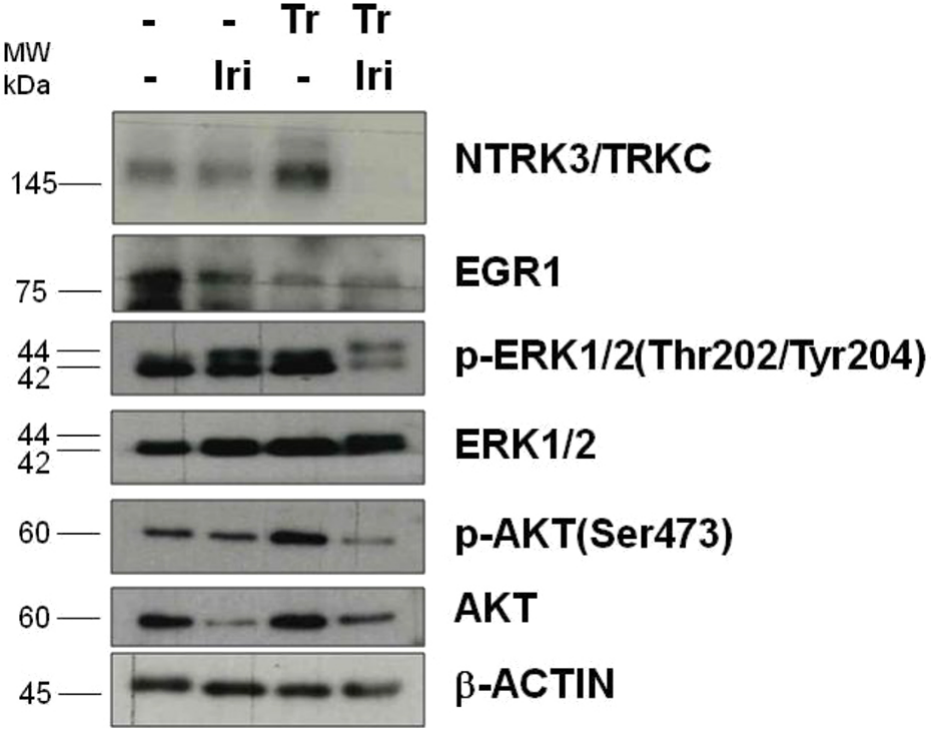
**Effects of irinotecan or trabectedin, as single agents or in combination, on *EWSR1::WT1* target gene expression.** Western blot analysis was performed on tumors excised after the first round of individual or combined treatment. Cropped images of selected proteins are shown in the different blots. Iri, irinotecan; Tr, trabectedin.

The trabectedin-irinotecan combination also affected the activity of the *EWSR1::WT1* fusion, as demonstrated by the downregulation of target genes, such as *NTRK3* (Ogura et al., 2021) and *EGR1* (Liu et al., 2000), which we observed at the protein level (Fig. 7). This reduction of the NTRK3 protein is paralleled by reduced gene transcription [log2(FC)=−0.4; nominal *P*=0.06]. In addition, the combined treatment reduced the expression of p-ERK1/2 (also known as MAPK3/1) (Thr202/Tyr204) and p-AKT (Ser473), suggesting the inhibition of RAS-MAPK and PI3K-AKT pathways, which are usually upregulated in DSRCT tumors (Smith et al., 2022).

## DISCUSSION

This study exploited a newly developed PDX of DSRCT harboring the *EWSR1::WT1* fusion to perform a comparative assessment of cytotoxic and targeted anticancer agents, focusing on irinotecan-based combinations. Irinotecan plus trabectedin was found to be the most effective treatment, apparently by interfering with cell cycle progression at the G2/M checkpoint and by inducing programmed cell death.

PDXs are translatable preclinical models that retain the main characteristics of clinical tumors (Hamacher and Bauer, 2017; Bleijs et al., 2019). Indeed, our DSRTC-1 PDX maintained the histomorphology of the paired clinical tumor, expressed the pathognomonic *EWSR1::WT1* gene fusion, showed some of the most frequent genomic aberrations observed in clinical DSRCT, including gains in chromosomes 1 and 3, and was characterized by a transcriptomic profile largely reproducing that of the originating tumor. PDXs appear particularly suitable for directly comparing standard-of-care agents and newer therapies in tumors, such as DSRCT, for which the level of clinical evidence for existing therapies is low, and comparative prospective clinical trials are lacking mainly because of disease rarity. Owing to the paucity of experimental models, in the past decade, preclinical pharmacological studies on DSRCT have been mainly performed following xenotransplantation of the JN-DSRCT-1 cell line into immunocompromised mice (Nishio et al., 2002). Only recently, DSRCT PDXs have been generated and used to evaluate the therapeutic potential of NTRK3 (Ogura et al., 2021) and EGFR (Smith et al., 2022) inhibitors for this disease.

To the best of our knowledge, this is the first study to compare the activity of cytotoxic and targeted drugs with different mechanisms of action when administered alone or in combination in DSRCT PDX. For our experiments, we identified anticancer drugs currently available as single agents for soft-tissue sarcomas and also relevant for DSRCT, with the final aim of exploring new combinations in comparison to monotherapy and providing preclinical data that could be instrumental in designing new clinical trials. In the DSRCT-1 PDX model, single-agent doxorubicin, larotrectinib, pazopanib and trabectedin showed modest antitumor activity and only caused tumor growth delay, although to a variable extent and without evidence of complete tumor regression. Conversely, when administered as single agents, vinorelbine, eribulin and irinotecan induced progressive tumor regression, with almost complete inhibition of tumor volume at the end of treatment, although afterwards all tumors started to regrow.

Interestingly, enhanced therapeutic efficacy was observed when irinotecan was combined with eribulin or trabectedin, resulting in complete objective responses in all treated mice. Tumor complete remission was maintained in all animals until 46 days after the end of irinotecan-eribulin treatment; mice treated with irinotecan plus trabectedin had no evidence of palpable tumors at the end of the experiment (i.e. 86 days after the last treatment). Histological analysis performed in tumors from sacrificed mice to confirm pathological complete response showed the presence of residual tumor cells in two of them, although proliferating cells were detected by Ki67 staining in only one tumor.

The combination of irinotecan and eribulin was tested in several PDXs of pediatric tumors, including rhabdomyosarcoma (RMS), Wilms tumor and rhabdoid tumor, along with cell line-derived xenografts of Ewing sarcoma (Robles et al., 2020). In this preclinical study, irinotecan was more effective when combined with eribulin compared to vincristine in six of 12 models, possibly through activation of the P53 pathway, with increased nuclear P53 accumulation and activation of apoptosis. A sequential treatment with trabectedin followed by irinotecan was tested in some patients with Ewing sarcoma and translocation-positive sarcomas, including a patient with a DSRCT, although outcomes for this patient are not specifically reported (Herzog et al., 2016). Interestingly, supported by the preclinical data here reported and the availability of trabectedin for the treatment of adult sarcomas, we have treated, at our Institution, two young adult patients with an advanced and heavily pretreated DSRCT with this combination, which resulted in a mixed and a complete response, as we recently reported (Ferrari et al., 2022b). Vinorelbine in combination with low-dose cyclophosphamide did obtain disease stabilization in DSRCT. The demonstration of the efficacy of this combination as maintenance therapy for high-risk RMS (Casanova et al., 2004; Bisogno et al., 2019) strengthens the possible value of this drug schedule for DSRCT, which was previously suggested for this specific tumor (Ferrari et al., 2007). Retrospective studies on antiangiogenic tyrosine kinase inhibitors in DSRCT suggest limited activity for sunitinib, sorafenib and apatinib, with few-dimensional responses and a median progression-free survival <6 months (Frezza et al., 2014a; Betrian et al., 2017; Shi et al., 2018), consistent with preclinical evidence for pazopanib in our DSRCT-1 PDX.

We also investigated the anticancer activity of larotrectinib, following a preclinical indication that the *EWSR1::WT1* fusion directly activates NTRK3 expression in DSRCT cells and that the NTRK inhibitor entrectinib may reduce the growth of DSRCT cells both *in vitro* and *in vivo* (Ogura et al., 2021). However, in our PDX model, larotrectinib showed modest antitumor activity. These data should be viewed together with other studies showing that the activity of NTRK inhibitors may be limited to tumors harboring a translocation involving NTRK rather than tumors with other NTRK rearrangements (Cocco et al., 2018) and the lack of NTRK fusions, such as DSRCT (Cocco et al., 2018; Okamura et al., 2018; Slotkin et al., 2021). Overall, current evidence does not support the use of this class of compounds in DSRCT.

Our study also provides valuable information on the cellular bases of the positive interaction between irinotecan and trabectedin or eribulin. Specifically, these drugs produced a cooperative effect, resulting in the complete abrogation of proteins involved in the G2/M checkpoint, suggesting cell arrest in the G2 phase, which was corroborated by the reduced expression of the mitotic marker p-H3 (Ser10). These drug combinations also markedly reduced the expression of antiapoptotic proteins and caused caspase-3 cleavage, consistent with the induction of apoptosis. Moreover, the combined treatment induced the accumulation of p-RIP1 (Ser166) and p-RIP3 (Ser227), indicating the occurrence of necroptosis (Gong et al., 2019), a type of programmed cell death with necrotic morphology. These findings are consistent with histomorphological analysis of tumors explanted from mice treated with irinotecan and trabectedin/eribulin combinations.

To gain insight into the molecular basis of the marked antitumor activity observed in our DSRCT PDX after irinotecan plus trabectedin, we performed a transcriptomic analysis in tumors explanted from mice exposed to single-agent irinotecan or trabectedin, or their combination. Irinotecan resulted in the most relevant reprogramming of tumor transcriptome compared to trabectedin, and the addition of trabectedin to irinotecan enhanced changes in the transcriptomic profile of the PDX, particularly the downregulation of gene sets related to cell cycle progression and proliferation. These findings, together with results from immunohistochemistry and western blot analysis, supported reduction of DSRCT proliferation potentially consequent to the arrest of tumor cells in the G2 phase of the cell cycle. This combination was also found to downregulate the activity of the *EWSR1::WT1* fusion and to counteract the abnormal activation of RAS-MAPK and PI3K-AKT signaling pathways.

The main limitation of our study is the availability of a single DSRCT PDX model, which limited the possibility of assessing response to drugs across different DSRCTs as well as investigations on modifiers of tumor response. In addition, the molecular basis underlying the potential synergistic effect of irinotecan-based combinations is far from being fully understood. However, DSRCT is an ultra-rare and incurable malignancy for which effective disease-specific and evidence-based treatment options are eagerly awaited. The information here generated, together with evidence from early clinical reports (Bisogno et al., 2021; Ferrari et al., 2022a,b; Verret et al., 2017; Brunetti et al., 2014; Lopez-Gonzalez et al., 2011; Emambux et al., 2017), are relevant for generating hypothesis to be tested in clinical studies.

This study emphasizes the importance of patient-derived preclinical models to explore new treatments in DSRCT. Our findings strongly support further investigations on the combination of irinotecan with either trabectedin or eribulin. The irinotecan-trabectedin combination appears to be particularly promising and deserves to be investigated in prospective clinical studies on relapsing DSRCT but also in chemotherapy-naïve patients, as the vast majority of DSRCT patients cannot be cured despite a very intensive chemotherapy regimen. Our data are also interesting from a drug development prospective as lurbinectedin, a novel synthetic agent derived from trabectedin with a similar mechanism of action, is currently under investigation in combination with irinotecan in sarcomas (NCT02611024).

## MATERIALS AND METHODS

### Patient and human tumor characterization

A 16-year-old male patient with a histopathological and molecular diagnosis of DSRCT underwent four cycles of irinotecan, ifosfamide, vincristine and actinomycin-D (IrIVA), which induced partial tumor response at the preoperative radiological assessment (Ferrari et al., 2022b). Surgery included a multivisceral resection with excision of multiple peritoneal nodules. At diagnosis, the neoplasm showed demarcated nests of small round cells within a desmoplastic stroma, for which spindle-shaped fibro-myofibroblasts were detected within a matrix of myxoid extracellular material and collagen. IHC showed immunoreactivity for WT1 (C19) in the absence of WT1 (180) reactivity. FISH analysis showed *EWSR1::WT1* translocation. Surgical specimens showed post-treatment changes such as fibro-histiocytic reaction and necrosis, which was surrounded by a combination of viable tumor cells and apoptotic cells.

### PDX development

The use of patient material to generate the PDX model was approved by the institutional ethical committee (INT-139/17). Generation of PDX and *in vivo* drug experiments were authorized by the Italian Ministry of Health (project approval code 234/2018-PR) and were performed in compliance with international policies and guidelines.

Fresh post-treatment DSRCT specimens were collected immediately after surgical resection, aseptically dissected and cut into small (∼3 mm^3^) fragments. At least three fragments were grafted subcutaneously into the right flank of 6-week-old female CB17/lcr-*Prkdc^scid^* (SCID) mice (Charles River Laboratories, Calco, Italy). Tumor growth was followed by bi-weekly measurement of tumor diameters with a Vernier caliper, and tumor volume (TV) was calculated according to the formula
(1)

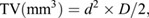
where *d* and *D* are the shortest and the longest diameters, respectively. After the third passage in mice, the PDX was considered established. We xenotransplanted DSRCT clinical tumors from two patients, and the PDX used in the study is the only one successfully established.

SCID mice were maintained in a pathogen-free facility, in which temperature and humidity were kept constant, and had free access to food and water. Mouse weight was routinely monitored during and after drug treatment for the whole experiment.

### PDX characterization

The consistency of the DSRCT-1 PDX with the originating clinical tumor was assessed in terms of histomorphology, presence of the *EWSR1::WT1* fusion, and genomic and transcriptomic profile.

### Histopathological analysis

Four-micrometer sections of formalin-fixed, paraffin-embedded (FFPE) tumor tissue obtained from the patient's surgical specimen and sacrificed mice were stained with Hematoxylin and Eosin (H&E) for morphological evaluation, and with antibodies against WT1 (C19) (1:1500, rabbit polyclonal antiserum, Thermo Fisher Scientific) and MIB1 (1:400, Ab Ki67, Clone Mib1, Dako). IHC analysis was performed at room temperature on a Dako Autostainer Link 48 AS480 (Agilent, Santa Clara, CA, USA), as previously described (Stacchiotti et al., 2019b). Ki67-labeling index, measured as the number of Ki67-positive nuclei/overall number of nuclei×100, was quantified using ImageJ 1.47q software. Pathologists with expertise in soft-tissue tumors compared the morphological features of human and PDX tumors during H&E and IHC analysis.

### FISH analysis

FISH was carried out to assess *EWSR1* and *WT1* gene status. *EWSR1* was evaluated using a commercial break-apart probe (LSI EWSR1 Dual Color Break Apart Rearrangement FISH Probe, Abbott Molecular), and *WT1* was assessed using an in-house made break-apart probe made up with the bacterial artificial chromosome (BAC) clones (Children's Hospital Oakland Research Institute, Oakland, CA, USA) RP11-299P16, covering the 5′ end of the gene, and RP11-259N9, covering the 3′ end. BACs were labeled with Spectrum Orange dUTP and Spectrum Green dUTP (Abbott Molecular) by means of nick translation (NICK translation Reagent Kit, Abbott Molecular), according to the manufacturer's instructions, and validated on normal metaphase spreads. FISH procedure for FFPE samples followed standard protocols. FISH slides were analyzed with a Leica DM 6000B (Wetzlar, Germany) microscope at 100× magnification and the appropriate fluorescence filters, and images were captured using Cytovision software (v. 7.0, Leica).

### Genomic profile

DNA was isolated from FFPE DSRCT clinical and paired PDX sample using a GeneRead DNA FFPE kit (Qiagen, Hilden, Germany) following the manufacturer's instructions. DNA was quantified using a Qubit dsDNA High-Sensitivity Assay Kit (Thermo Fisher Scientific), and DNA quality was assessed by TapeStation 4200 (Agilent Technologies). The OncoScan™ CNV Plus Assay (Thermo Fisher Scientific) was implemented following the manufacturer's instructions to detect genome-wide copy number gains and losses, LOH, including copy neutral LOH, and a panel of somatic mutations. Data were visualized with the karyoploteR tool implemented in R (Gel and Serra, 2017).

### Transcriptomic profile

Total RNA was extracted from frozen DSRCT clinical and PDX specimens with an RNeasy mini kit (Qiagen) following the manufacturer's instructions. The concentration and the purity of the RNA starting material were measured on a spectrophotometer, and RNA integrity number (RIN) was measured on an Agilent TapeStation 4200 (RNA ScreenTape Assay, Agilent Technologies).

For the clinical tumor and paired DSRCT-1 PDX, cDNA libraries were synthesized from 200 ng total RNA with an RNA Prep kit with Tagmentation (Illumina, Agilent Technologies), following the manufacturers' instructions. Libraries were quantified by Qubit assay (Thermo Fisher Scientific), and quality was assessed by the High Sensitivity kit on the 4200TapeStation (Agilent Technologies). Sequencing was performed at 2×75 bp using an Illumina NextSeq500 sequencer.

Total RNA extracted from untreated PDXs and PDX exposed to one round of treatment with irinotecan or trabectedin as a single agent or in combination was processed according to a standard NEBNext^®^ Ultra™ II Directional RNA Library Prep Kit (New England Biolabs, Ipswich, MA, USA) for Illumina^®^ protocol, and mRNA was selected with oligo-dT beads starting from 800 ng total RNA. Libraries were quantified and quality checked on an Agilent 4200 TapeStation system (High Sensitivity D5000, Agilent Technologies) and then sequenced on an Illumina NovaSeq platform with paired-end reads 150 bp long, with a depth of 27 million clusters/sample.

After the quality control performed with the fastQC tool, sequences were aligned using STAR against human reference genome hg19. Read counts were determined according to the protocol used for library preparation. Raw data were normalized using the trimmed mean of M-value, according to the edgeR package (Robinson et al., 2010) of the R environment, and filtered, discarding reads under the tenth percentile of expression variance across samples and lacking an associated official gene symbol. Reads mapping on the same gene symbol were collapsed, summing their values. Gene expression data and the preprocessing pipeline were deposited at Gene Expression Omnibus (GEO; accession number GSE220172). Correlation between PDX and clinical tumor gene expression profiles was evaluated with the Spearman coefficient in order to detect a possible non-linear relationship. Differential expression analysis was performed by removing data heteroscedasticity with the voom method implemented into the edgeR package (Robinson et al., 2010). A linear model was employed to evaluate differentially expressed genes using the limma package (Ritchie et al., 2015), measuring both the fold change (FC), logarithmically (base 2) transformed, and the *t*-statistic. Taking into account multiple comparisons to assess significant changes, a threshold of 0.05 was considered for the false discovery rate (FDR) *P*-value correction. Gene set enrichment analysis was performed on the hallmark collection of the Molecular Signatures Database, and genes were ranked according to the *t*-statistic as implemented into the fgsea package (Korotkevich et al., 2019 preprint). An FDR threshold of 0.05 was applied to assess significant enrichments.

### Metadataset analysis

Normalized transcriptomic data of two publicly available datasets [GEO accession number GSE90904; http://cbio.mskcc.org/public/sarcoma_array_data/filion2009.html (Smith et al., 2022)] and our series of PDXs with the paired clinical tumors of different soft tissue sarcoma histology were gathered together into a metadataset. Batch effects were removed by applying the ComBat algorithm implemented in the sva package (Leek et al., 2012). The non-linear t-SNE statistical method was employed to visualize high-dimensional data in a low-dimensional space.

### Drug activity studies

When tumor burden reached ∼150-200 mm^3^ (∼60 days after small fragment implant), SCID mice were randomized to receive different drugs. Each experimental group consisted of nine mice; six mice were used to assess drug effect on tumor growth over time, and three mice were sacrificed after the first round of each individual or combined treatment to carry out H&E, IHC, western blot and RNA-sequencing analyses.

Three independent experiments were performed. In the first one, mice were treated with single-agent doxorubicin (Adriblastina, Accord Healthcare, Milan, Italy), pazopanib (Med Chem Express, D.B.A, Milan, Italy), larotrectinib (Vitrakvi, Bayer, SpA, Milan, Italy) and vinorelbine (Navelbine, Pierre Fabre Pharma Srl, Milan, Italy). In the second experiment, SCID mice received eribulin (Halaven, EISAI Srl, Milan, Italy) and irinotecan (Accord Healthcare), administered singly or in combination. In the third experiment, SCID mice were treated with irinotecan and trabectedin (Yondelis, PharmaMar, Peschiera Borromeo, Italy), alone or in combination. After dilution in sterile water (doxorubicin, trabectedin), saline solution (eribuline, irinotecan, vinorelbine) or 0.5% methyl cellulose (pazopanib, larotrectinib), drugs were administered at dosage schedules reported in Table 1. The same doses and schedules were used when drugs were administered as single agents and in combination.

Drug activity was evaluated as (1) tumor volume inhibition (TVI) percentage using the formula
(2)

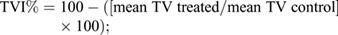
and (2) complete tumor regression, defined as tumor disappearance, evaluated by palpability, lasting for at least 10 days during or after treatment. Treatment toxicity was determined in terms of body weight loss and lethal toxicity.

### Western blotting

Total proteins were extracted from frozen PDX tumors after the first round of treatment at different times after the start of treatment as described previously (Stacchiotti et al., 2019b). Tumor lysates were separated by SDS-PAGE and transferred onto nitrocellulose membranes. The membranes were incubated overnight at 4°C with the primary antibodies: anti-CDK1 (1:4000, sc-53, Santa Cruz Biotechnology, Beverly, MA, USA), anti-CDC25A (1.2000, sc-79, Santa Cruz Biotechnology), anti-BAX (1:1000, sc-493, Santa Cruz Biotechnology), anti-BCL2 (1:1000, sc-509, Santa Cruz Biotechnology), anti-EGR1 (1:1000, 4154, Cell Signaling Technology), anti-p-CDK1 (Tyr 15) (1:1000, 9111, Cell Signaling Technology), anti-p-H3 (Ser10) (1:1000, 9062, Cell Signaling Technology), anti-PLK1 (1:1000, 4513, Cell Signaling Technology), anti-survivin (1:2000, ab469, Abcam, Cambridge, UK), anti-P53 (1:1000, sc-126, Santa Cruz Biotechnology Inc), anti-HMGB1 (1:1000, 6893, Cell Signaling Technology), anti-HMGA1 (1:1000, 12094, Cell Signaling Technology), anti-cleaved caspase 3 (1:1000, 9661, Cell Signaling Technology), anti-RIP3 (1:1000, 13526, Cell Signaling Technology), anti-p-RIP3 (Ser227) (1:1000, 93654, Cell Signaling Technology), anti-RIP1 (1:1000, 65746, Cell Signaling Technology), anti-p-RIP1 (Ser166) (1:1000, 3493, Cell Signaling Technology), anti-NTRK3 (1:1000, 3376, Cell Signaling Technology), anti-p-p44/42 MAPK (Erk1/2) (Thr202/Tyr204) (1:1000, 9101, Cell Signaling Technology), anti-p44/42 MAPK (ERK1/2) (1:1000, 4695, Cell Signaling Technology), anti-p-AKT (Ser473) (1:1000, 9271, Cell Signaling Technology) and anti-AKT (1:1000, 310861, BD Transduction Laboratories). Anti-rabbit or anti-mouse secondary peroxidase-linked whole antibodies (Cell Signaling Technology) were used to detect primary antibody.

The membranes were cut to allow simultaneous incubation of primary antibodies detecting proteins with different molecular mass on the same membrane. Membranes were stripped and reincubated with different primary antibodies and successively with the specific peroxidase secondary antibodies. For the preparation of figures, we cropped original film western blot images to generate a unique panel with different proteins. Molecular masses were determined using Precision Plus Protein™ Standard (Bio-Rad Laboratories, Hercules, CA, USA), which yields a colorimetric image only and has been removed from the chemoluminescence blot image.

### Statistical analysis

Statistical analysis was performed with Wilcoxon rank-sum test and with Kruskal–Wallis test, when appropriate, using GraphPad Prism software (version 9.4; GraphPad Prism Inc., San Diego, CA, USA) and STATA/IC (version 15.1; Stata Corp LLC, College Station, TX, USA). *P*≤0.05 was considered statistically significant.

## Supplementary Material

10.1242/dmm.049649_sup1 Supplementary information
