# Supplementary material for: Effectiveness of irinotecan plus trabectedin on a desmoplastic small round cell tumor patient-derived xenograft
Source: Dis Model Mech. 2023 Jun 14;16(6):dmm049649. doi: 10.1242/dmm.049649 (PMC10281256; doi:10.1242/dmm.049649)
Supplement: Supplementary information [file dmm-16-049649-s1.pdf]

A

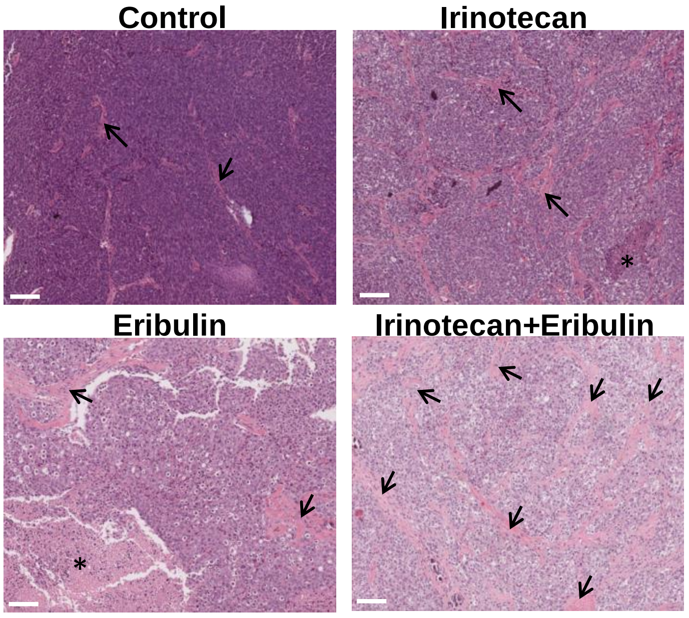

	Sclerojalinosis (%) →	Necrosis (%) *
**Control**	5	10
**Eribulin**	10	25
**Irinotecan**	15	15
**Irinotecan+ Eribulin**	35	10

B

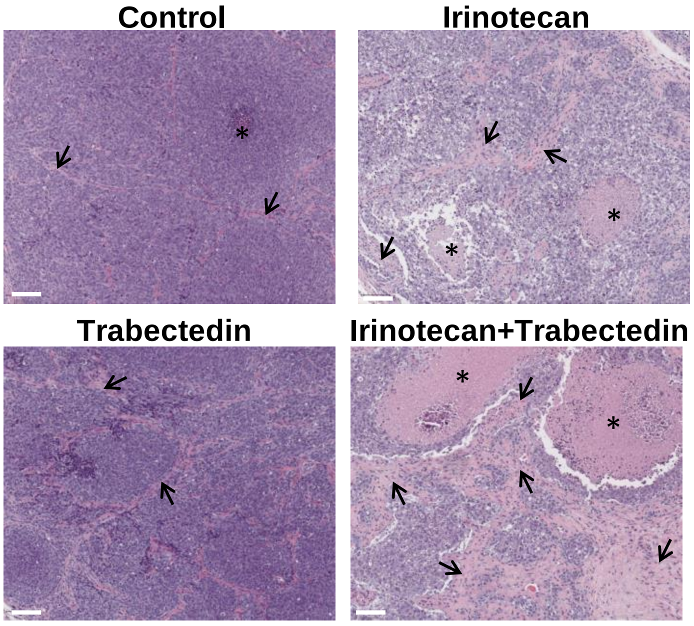

	Sclerojalinosis (%) →	Necrosis (%) *
**Control**	5	5
**Irinotecan**	20	15
**Trabectedin**	10	5
**Irinotecan+ Trabectedin**	40	25

Fig. S1. Morphological changes after treatment with irinotecan alone or combined with (A) eribulin or (B) trabectedin. Representative images of H&E-stained DSRCT-1 PDX tissue sections at the appropriate magnification to exhibit the different presence of sclerojalinosis (→) and necrotic (*) areas induced by the different treatments. In table are reported necrotic and slerojalinosis area percentages to total area. Scale bar: 100μm.

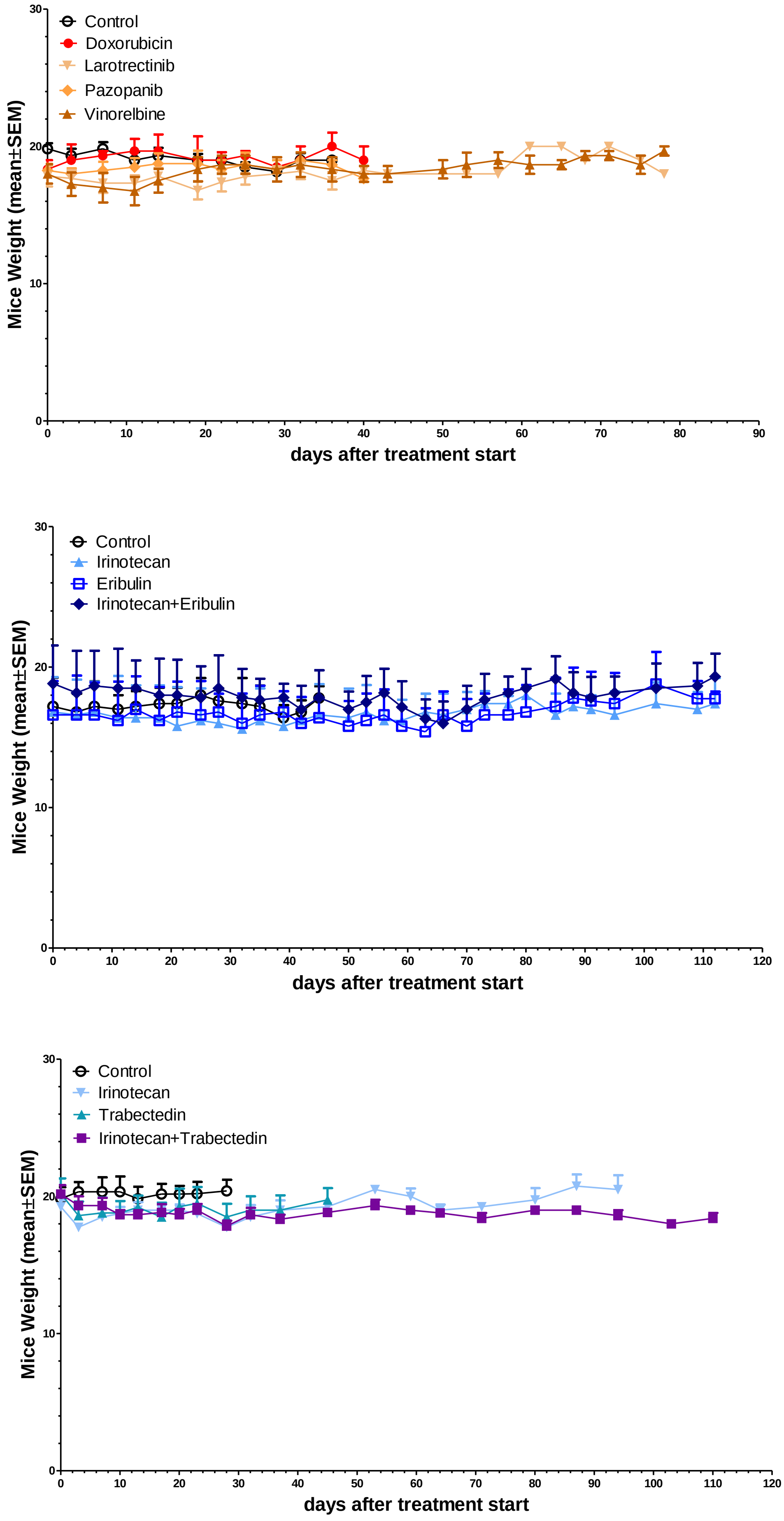

Fig. S2. Mouse weight modulations during the course of different experiments with single agents and drug combinations.

Disease Models & Mechanisms • Supplementary information

	ctrl	Doxorubicin	Pazopanib
Pospho-H3 (Ser10)	1	0,7	0,8
Pospho-CDK1 (Tyr15)	1	1,6	1,2
CDK1	1	1,8	1,9
PLK1	1	1,6	1,8
CDC25A	1	1,2	1,4
SURVIVIN	1	2,4	2,9
P53	1	1,1	1,4
HMGB1	1	2,2	2,3
HMGA1	1	1,2	3,1
BAX	1	1,9	2,0
BCL2	1	1,3	2,1
CLEAVED CASPASE 3	0	0,0	0,0
Pospho RIP (Ser166)	1	1,1	2,3
RIP1	1	2,2	1,8
Pospho RIP (Ser227)	1	5,2	0,0
RIP3	1	1,2	1,0

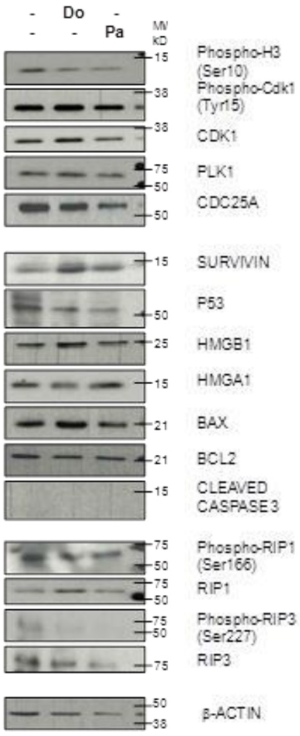

	ctrl	Larotrectinib
Pospho-H3 (Ser10)	1	1,5
Pospho-CDK1 (Tyr15)	1	1,1
CDK1	1	1,7
PLK1	1	1,0
CDC25A	1	1,0
SURVIVIN	1	1,3
P53	1	0,9
HMGB1	1	1,2
HMGA1	1	1,2
BAX	1	0,8
BCL2	1	1,1
CLEAVED CASPASE 3	0	0,0
Pospho RIP (Ser166)	1	1,1
RIP1	1	0,7
Pospho RIP (Ser227)	1	1,2
RIP3	1	0,8

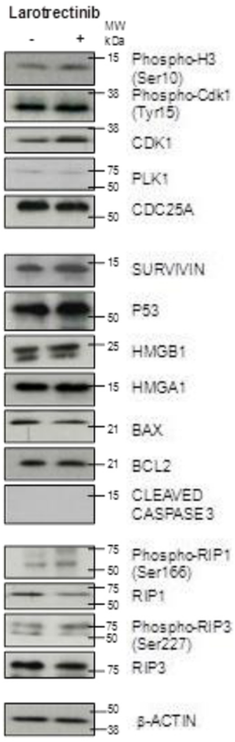

	ctrl	Vinorelbine
Pospho-H3 (Ser10)	1	17,2
Pospho-CDK1 (Tyr15)	1	2,1
CDK1	1	1,3
PLK1	1	5,7
CDC25A	1	0,8
SURVIVIN	1	2,5
P53	1	0,7
HMGB1	1	1,0
HMGA1	1	0,2
BAX	1	0,5
BCL2	1	0,3
CLEAVED CASPASE 3	0	0,0
Pospho RIP (Ser166)	1	1,5
RIP1	1	0,3
Pospho RIP (Ser227)	1	1,9
RIP3	1	0,7

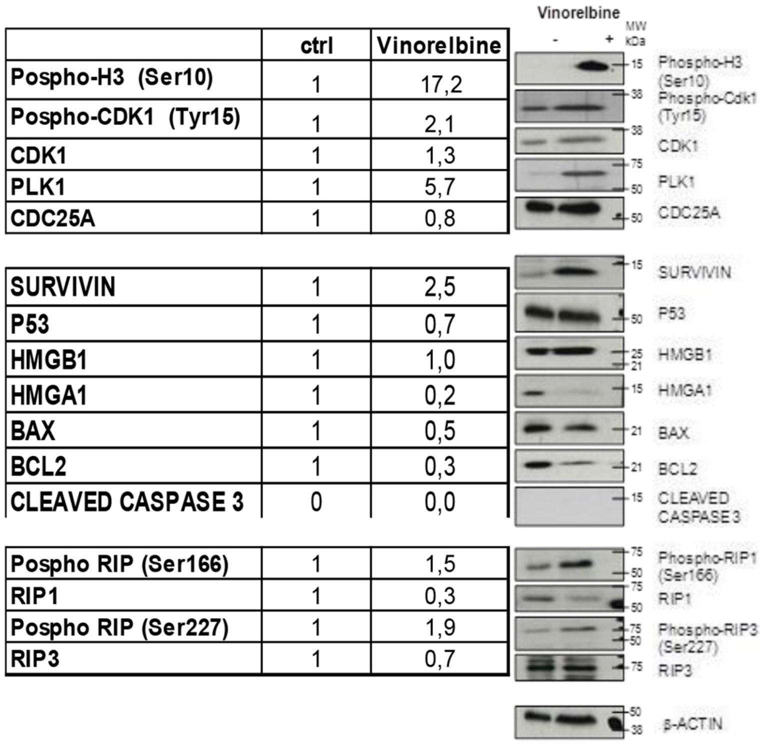

	ctrl	Irinotecan	Eribulin	Iri+Eri
Pospho-H3 (Ser10)	1	3,6	0,6	0,0
Pospho-CDK1 (Tyr15)	1	0,7	0,9	0,3
CDK1	1	1,0	0,8	0,1
PLK1	1	0,7	0,4	0,1
CDC25A	1	0,7	1,0	0,6
SURVIVIN	1	1,8	1,4	0,3
P53	1	1,0	0,6	0,5
HMGB1	1	0,8	0,9	0,1
HMGA1	1	0,4	0,4	0,0
BAX	1	0,9	1,1	0,9
BCL2	1	0,9	1,3	0,8
CLEAVED CASPASE 3	1	1,2	9,0	10,3
Pospho RIP (Ser166)	1	0,6	1,1	1,6
RIP1	1	0,7	0,8	0,9
Pospho RIP (Ser227)	1	0,9	0,6	2,5
RIP3	1	0,6	0,8	0,6

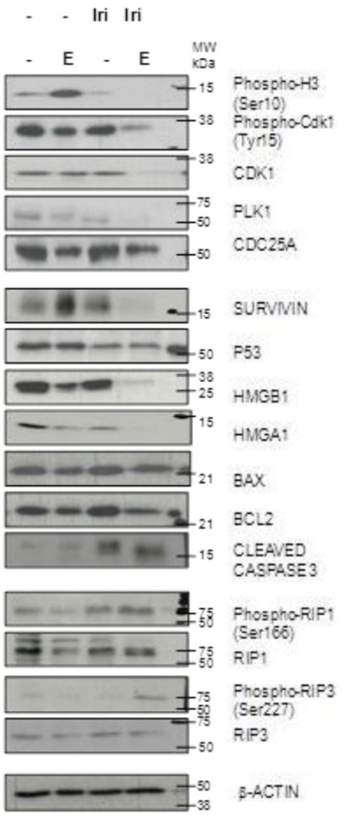

	ctrl	Irinotecan	Trabectidin	Iri+Trabe
Pospho-H3 (Ser10)	1	0,8	0,5	0,0
Pospho-Cdk1 (Tyr15)	1	1,0	0,5	0,2
CDK1	1	1,0	0,8	0,1
PLK1	1	1,4	1,3	0,1
CDC25A	1	1,2	1,5	0,1
SURVIVIN	1	1,5	1,2	0,3
P53	1	1,2	0,8	1,8
HMGB1	1	1,8	0,7	0,4
HMGA1	1	1,5	1,7	0,0
BAX	1	1,2	1,3	1,5
BCL2	1	2,3	1,4	0,1
CLEAVED CASPASE 3	1	1,3	3,1	9,7
Pospho RIP (Ser166)	1	2,5	1,2	7,5
RIP1	1	1,3	0,9	0,3
Pospho RIP (Ser227)	1	2,2	1,0	26,7
RIP3	1	1,4	0,4	0,5

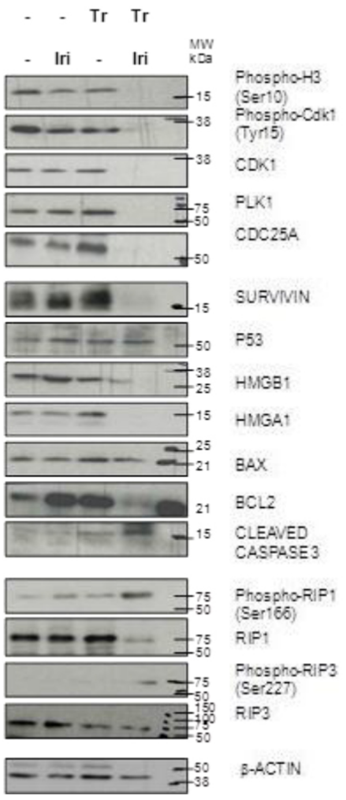

	ctrl	Irinotecan	Trabectidin	Iri+Trabe
TRKC	1	0,9	2,9	0,0
EGR1	1	1,1	0,8	0,6
p-ERK1/2(Thr202/Tyr204)	1	0,8	1,0	0,2
ERK1/2	1	1,3	1,7	1,8
p-AKT(Ser473)	1	0,8	2,3	0,6
AKT	1	0,1	0,9	0,2

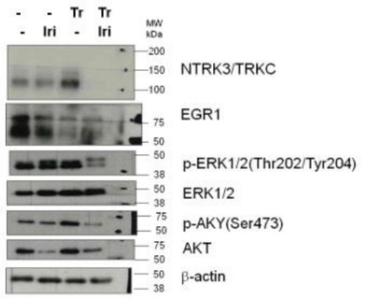

Fig. S3. Protein markers and quantification of band intensities for each blot reported in Fig. 5. Quantification values for each band were performed using ImageJ. After normalization to corresponding β-actin, normalized values for each band and each treatment were compared to the corresponding band of the untreated sample (-).

Disease Models & Mechanisms • Supplementary information

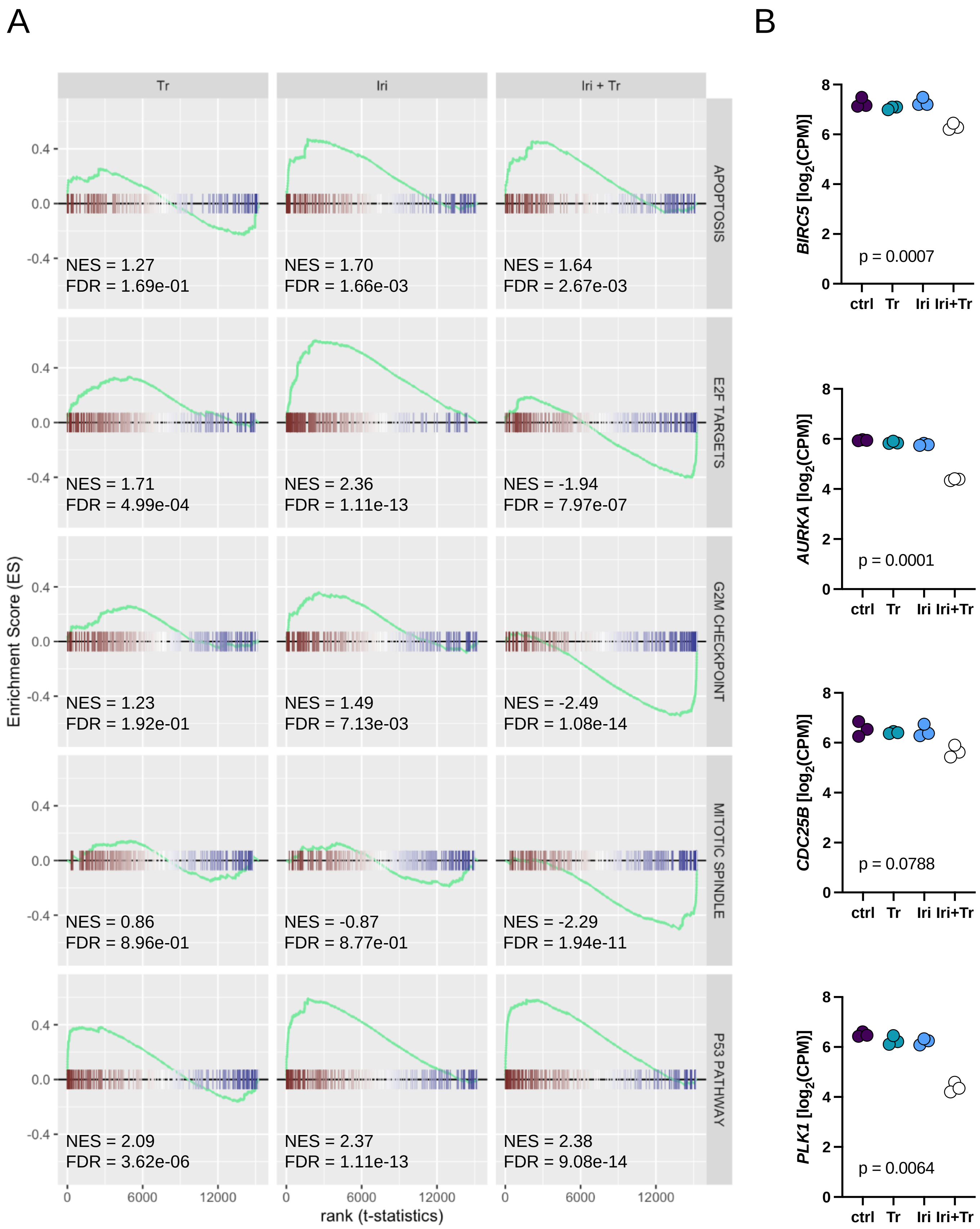

Fig. S4. Effects of irinotecan or trabectedin, as single agents or in combination, on relevant gene sets and selected genes. (A) Enrichment plot for relevant gene sets that mirror the heatmap in Figure 6F of the following comparisons: trabectedin (Tr) vs control, irinotecan (Iri) vs control, irinotecan plus trabectedin (Iri+Tr) vs control. **(B)** Expression of *BIRC5, AURKA, CDC25B*, and *PLK1* in control (ctrl) mice and in mice treated with trabectedin (Tr), irinotecan (Iri), or irinotecan plus trabectedin (Iri+Tr).

Disease Models & Mechanisms • Supplementary information

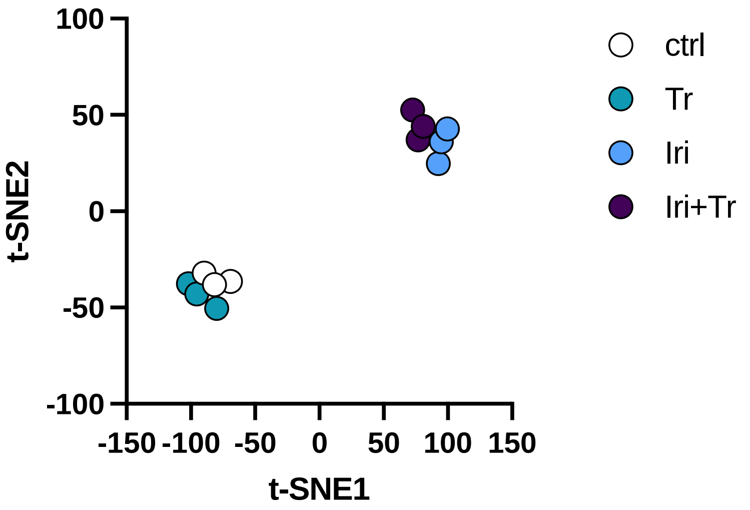

Fig. S5. Scatter plot of t-SNE of control DSRCT PDX (ctrl) as well as PDX treated with trabectedin (Tr), irinotecan (Iri), or irinotecan plus trabectedin (Iri+Tr).
